# Lingonberry (*Vaccinium vitis*-*idaea* L.) Skin Extract Prevents Weight Gain and Hyperglycemia in High-Fat Diet-Induced Model of Obesity in Mice

**DOI:** 10.3390/nu16132107

**Published:** 2024-07-02

**Authors:** Riitta Ryyti, Mari Hämäläinen, Tiina Tolonen, Marianne Mäki, Mari Jaakkola, Rainer Peltola, Eeva Moilanen

**Affiliations:** 1 The Immunopharmacology Research Group, Faculty of Medicine and Health Technology, Tampere University and Tampere University Hospital, 33014 Tampere, Finland; riitta.ryyti@tuni.fi (R.R.); mari.hamalainen@tuni.fi (M.H.); 2 Unit of Measurement Technology, Kajaani University Consortium, University of Oulu, 87400 Kajaani, Finland; tiina.tolonen@oulu.fi (T.T.); mariannemk82@gmail.com (M.M.); marde.jaakkola@gmail.com (M.J.); 3 Bioeconomy and Environment, Natural Resources Institute Finland, 96200 Rovaniemi, Finland; rainer.peltola@luke.fi

**Keywords:** lingonberry, high-fat diet, weight gain, obesity, blood glucose, insulin, polyphenol

## Abstract

The percentage of obese people is increasing worldwide, causing versatile health problems. Obesity is connected to diseases such as diabetes and cardiovascular diseases, which are preceded by a state called metabolic syndrome. Diets rich in fruits and vegetables have been reported to decrease the risk of metabolic syndrome and type 2 diabetes. Berries with a high polyphenol content, including lingonberry (*Vaccinium vitis*-*idaea* L.), have also been of interest to possibly prevent obesity-induced metabolic disturbances. In the present study, we prepared an extract from the by-product of a lingonberry juice production process (press cake/pomace) and investigated its metabolic effects in the high-fat diet-induced model of obesity in mice. The lingonberry skin extract partly prevented weight and epididymal fat gain as well as a rise in fasting glucose level in high-fat diet-fed mice. The extract also attenuated high-fat diet-induced glucose intolerance as measured by an intraperitoneal glucose tolerance test (IPGTT). The extract had no effect on the levels of cholesterol, triglyceride or the adipokines adiponectin, leptin, or resistin. The results extend previous data on the beneficial metabolic effects of lingonberry. Further research is needed to explore the mechanisms behind these effects and to develop further health-promoting lingonberry applications.

## 1. Introduction

Obesity is an increasing health problem, and it is now considered a worldwide epidemy. Obesity predisposes individuals to chronic diseases, particularly type 2 diabetes and cardiovascular diseases [1]. These are preceded by a state called metabolic syndrome. It includes several interconnected factors increasing the risk of atherosclerotic diseases, type 2 diabetes, and fatty liver disease: abdominal obesity, hypertension, dyslipidemia, insulin resistance, and dysregulated glucose homeostasis [2]. Diets rich in fruits and vegetables can help to decrease the risk of metabolic syndrome and its co-morbidities [3].

Lingonberry (*Vaccinium vitis*-*idaea* L.) is a commonly consumed wild-growing berry in the boreal areas of the Northern Hemisphere. It is rich in polyphenols and has a high antioxidant capacity [4,5,6,7]. Interestingly, lingonberry supplementation has been shown to prevent high-fat diet-induced weight gain in animal models [8,9,10]. Moreover, lingonberry supplementation has been reported to have a beneficial effect on blood glucose [8] and insulin [11] levels, as well as on lipids and inflammatory markers [8,10]. It remains, however, unclear which compounds in lingonberry contribute to these beneficial effects. Some preliminary data suggest that total polyphenol and antioxidant concentrations as well as proanthocyanidins, anthocyanins, resveratrol, and kaempferol may have a role in these effects [12,13,14].

A press cake (also called as pomace) is formed as a by-product in the berry juice production process. It contains the skin and seeds of the treated berries and includes biologically valuable phenolic compounds [15,16]. Here, our aim was to investigate the exploitation of the side streams of the industrial berry juice manufacturing process, and we prepared a lingonberry skin extract (LSE) from lingonberry press cake material where seeds had initially been removed. The content of the extract was analyzed with HPLC-based methods and its metabolic effects were investigated in an experimental model of obesity in mice. The extract had such positive effects on weight gain and glucose metabolism that warrant further studies experimentally and clinically.

## 2. Materials and Methods

### 2.1. Animals and Diets

Eight-week-old male C57BL/6N mice (Scanbur Research A/S, Karlslunde, Denmark) were included in the study. Mice were randomly allocated into three groups with twelve animals per group and housed under standard conditions (temperature 22 ± 1 °C, humidity 50–60%, and dark/light cycle 12/12 h) at Tampere University Preclinical Unit. One group of mice were given a low-fat diet (LF diet, 10% of energy from fat) and two groups a high-fat diet (HF diet, 46% of energy from fat) for six weeks (diets were obtained from Research Diets Inc, New Brunswick, NJ, USA).

The fat in the HF diet feed consisted of lard (20.7 g/100 g of feed) and soybean oil (2.9 g/100 g of feed), and the fatty acid composition of the feed was 32% of saturated, 35% of monounsaturated and 35% of polyunsaturated fatty acids of the total fat. The mice were dosed each weekday with vehicle (LF and HF diet groups) or lingonberry skin extract (HF + LSE diet group) 8.5 µL/g body weight by intragastric administration; the dosage was based on previous experience and recommended volumes. Food consumption and body weight of the mice were measured weekly.

The sample size (number of mice per group) was set based on our previous study [8] to detect an at least 2.0 mmol/L lower fasting glucose level in the LSE-supplemented HF diet-fed mice than in the mice on the control HF diet. Other parameters were determined as standard deviation (SD) of 1.5 mmol/L [8], and with a Type I significance level (alpha) of 0.05 and Type II significance level (beta) of 0.2. There were no drop-outs during the study.

An intraperitoneal glucose tolerance test (IPGTT) was carried out at week five, one week before the end of the experiment. In the IPGTT test, after a six-hour morning fast, animals received an intraperitoneal injection of glucose (2 g/kg body weight, Sigma-Aldrich, St. Louis, MO, USA), and circulating glucose concentrations were measured from the tail vein before and 30, 60, 120, and 180 min after the injection (Contour Next One, Oy Diabet Ab, Lemu, Finland).

When terminating the study, six-hour fasted animals were anesthetized with isoflurane, blood glucose was analyzed, and thereafter, blood and tissue samples were collected as previously described [8]. The amount of epididymal fat was weighed immediately after the sacrifice. Fluorometric assays were used to measure serum cholesterol and triglyceride concentrations (Abcam, Cambridge, UK). Insulin (Mercordia Ltd., Uppsala, Sweden), adiponectin, leptin, and resistin (R&D Systems Europe Ltd., Abingdon, UK) levels were analyzed with ELISA. The HOMA-IR values (homeostatic model assessment of insulin resistance) were calculated according to the previously published model [17]: fasting plasma insulin (mU/L, conversion factor adopted from [18]) was multiplied by fasting plasma glucose (mmol/L) and divided by the constant 22.5.

### 2.2. Lingonberry Skin Extract and Analysis of Phenolic Compounds

The extract used in the present study was prepared from lingonberry (*Vaccinium vitis-idaea* L.) juice press residue material, which was obtained from Kiantama Ltd. (Suomussalmi, Finland). The wet press residue, containing the skin parts and seeds of the berry, was first dried, and then the seeds were separated by sieving. Dried lingonberry skin fraction (150 g) was extracted with 800 mL of 28% ethanol (*v*/*v*) in an autoclave at 120 °C for 60 min. The phenolic composition and total antioxidant capacity of the extract was analyzed in the University of Oulu, Kajaani University Consortium, Kajaani, Finland.

For the analysis of total proanthocyanidins in the lingonberry ethanol extract, a colorimetric 4-dimethylaminocinnamaldehyde (DMAC) method was applied as published by Prior et al. [19]. The analysis was performed with Varioskan flash microplate reader (Thermo Scientific, Vantaa, Finland) using procyanidin A2 as the standard. The amount of quercetin and glycosides of quercetin as aglycons after acid hydrolysis (with 1 M HCl 1:1 *v*:*v*, 2 h in 70 °C) were measured by a method modified from our previous publication [20], using an Agilent (Agilent Technologies, Inc, Santa Clara, CA, USA) 1100 series high-performance liquid chromatography (HPLC) instrument equipped with a diode array detector (DAD) and Gemini C18 column (3 mm × 150 mm, 5 µm, Phenomenex). The mobile phase consisting of 1% formic acid in water (eluent A) and acetonitrile (eluent B) was used with linear gradients: 10–15% B (0–2 min), 15–25% B (2–15 min), 25–40% B (15–20 min), and 40–90% B (20–25 min). Quercetin as an external standard detected at the wavelength of 370 nm was applied for the quantification. Anthocyanins were measured with the Agilent HPLC instrument described above using modified method of Pap et al. [21]. A linear gradient of 10% formic acid (eluent A) and acetonitrile (eluent B) was run with Gemini C18 column (3 mm × 150 mm, 5 µm, Phenomenex). The gradients used in the flow rate of 0.4 mL/min were as follows: 5% B (0–2 min), 5–15% B (2–20 min), 15–17% B (20–22 min), and 17–90% B (22–25 min). Anthocyanins were identified in a wavelength of 520 nm by comparing retention times to our in-house bilberry juice control for anthocyanins. Concentrations of anthocyanins detected from lingonberry extract were calculated by applying a standard curve of cyanidin-3-glucoside. Benzoic and other phenolic acids were analyzed with Agilent HPLC instrument described above, with the method published by Tsiapara et al. [22]. A modified wavelength (λ) of 320 nm was applied for the quantitation of five phenolic acids, along with a λ of 260 nm from the original method, that was used for the other acids. A representative figure of each compound group analyzed with HPCL is presented in the Supplementary Materials. Total amount of phenolic compounds was measured according to Spilioti et al. [23] using the Folin–Ciocalteau method [24]. A modified method of Huang et al. [25] was used as described by Kallio et al. [26] to measure oxygen radical absorbance capacity (ORAC) of the lingonberry extract.

### 2.3. Statistics

Results are presented as the mean ± SEM (standard error of the mean). Repeated-measure one-way or two-way analysis of variance (ANOVA) followed by Bonferroni multiple-comparison test was used in the statistical analysis. Differences were considered significant at *p* < 0.05 and marked with asterisks, indicating differences: * = *p* < 0.05, ** = *p* < 0.01, *** = *p* < 0.001 and **** = *p* < 0.0001. Calculations were carried out with the Prism computerized package (Graph Pad Prism Software, versions 8 and 9, San Diego, CA, USA).

## 3. Results

### 3.1. Composition of the Extract

The extract was found to contain proanthocyanidins (1.19 mg/mL), quercetin and its glycosides (0.04 mg/mL), anthocyanins (0.46 µg/mL), benzoic acid (0.31 mg/mL), and minor amounts of protocatechuic, chlorogenic, cinnamic, and p-coumaric acids. The content of total phenolic compounds was 2.66 mg/mL, and antioxidant capacity (ORAC) was 39.76 µmol TE/mL.

### 3.2. Weight Gain

**Figure 1:**
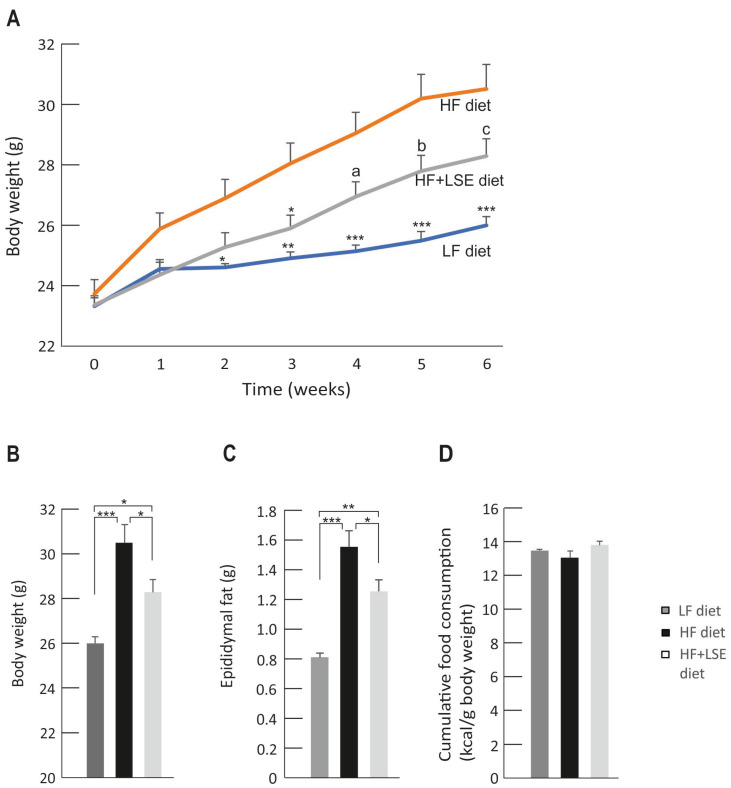
Weight gain and food consumption. Body weight development during the study (**A**), and body (**B**) and epididymal fat (**C**) weight at the end of the study. The results are presented in grams (g). The cumulative food intake during the study, expressed as energy unit per body weight (kcal/g) (**D**). Low-fat diet (LF diet, 10% of energy from fat) or high-fat diet (HF diet, 46% of energy from fat) was given for the animals for six weeks. Mice were dosed each weekday with vehicle (LF and HF diet groups) or lingonberry skin extract (HF + LSE diet group) 8.5 µL/g body weight by intragastric administration. Repeated-measures two-way ANOVA with Bonferroni post-test was applied in the statistical analysis (**A**). The values in (**B**–**D**) were analyzed with one-way ANOVA and Bonferroni post-test. Mean values significantly different from the HF diet group are marked with * = *p* < 0.05, ** = *p* < 0.01, and *** = *p* < 0.001, a = 0.068, b = 0.069, and c = 0.113. Values are given as the mean + SEM, *n* = 12 mice per group.

At the onset of the study, the weight of the mice was 23.5 ± 0.4 g, and there was no difference between the groups. The weight of the mice increased significantly in the high-fat (HF) diet group during the study when compared to the low-fat (LF) diet group (*p* < 0.001) as shown in Figure 1A. Notably, lingonberry skin extract supplementation partly prevented the HF diet-induced weight gain, as the weight of the mice in the HF + LSE diet group was significantly lower than in the HF diet group (*p* < 0.05 between the HF and HF + LSE diet groups). At the close of the study, the weights of the mice were 30.5 ± 0.8 g in the HF diet group, 28.3 ± 0.6 g in the HF + LSE diet group, and 26.0 ± 0.3 g in the LF diet group (Figure 1B).

Moreover, the amount of epididymal fat was higher in the HF diet group (1.6 ± 0.1 g) than in the LF diet group (0.8 ± 0.03 g), with *p* < 0.001 between the groups. Lingonberry skin extract significantly prevented the HF diet-induced accumulation of epididymal fat, as the amount of epididymal fat (1.3 ± 0.1 g) was significantly lower in the HF + LSE diet group when compared to the HF diet group (*p* < 0.05) (Figure 1C). Food consumption was followed weekly, and the cumulative food intake did not differ between the groups, expressed as kcal/g body weight (Figure 1D).

### 3.3. Glucose, Insulin, and Glucose Tolerance Test

**Figure 2:**
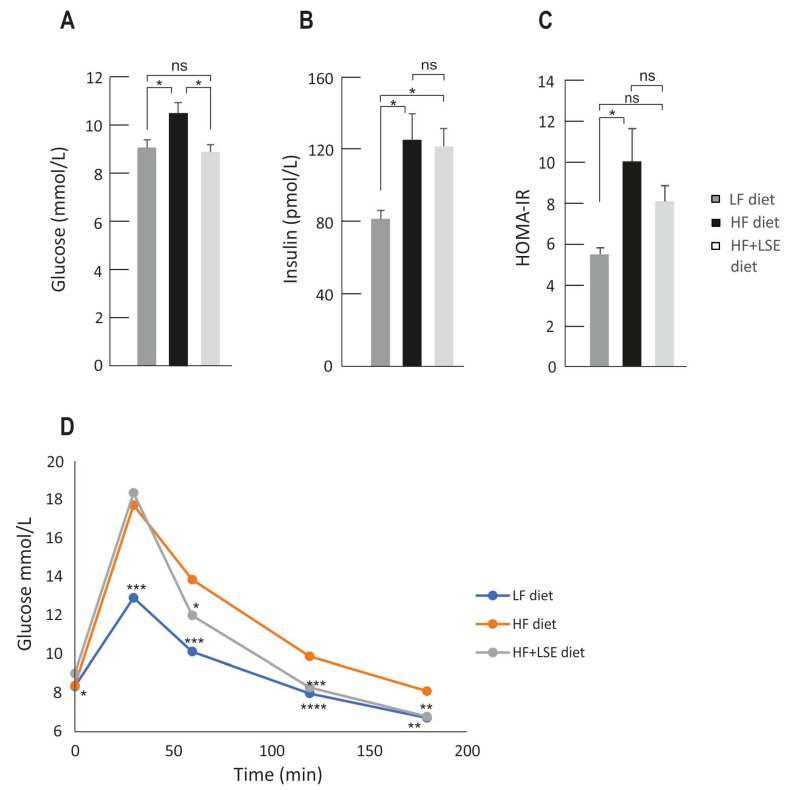
The fasting blood glucose (**A**) and insulin (**B**) levels and the HOMA-IR index (**C**) of the mice at the end of the study, and an intraperitoneal glucose tolerance test (IPGTT) one week before the end of the experiment (**D**). Animals received low-fat diet (LF diet, 10% of energy from fat) or high-fat diet (HF diet, 46% of energy from fat) for six weeks. Mice were dosed each weekday with vehicle (LF and HF diet groups) or lingonberry skin extract (HF + LSE diet group) 8.5 µL/g body weight by intragastric administration. In the IPGTT test (**D**), the animals received glucose (2 g/kg body weight) by intraperitoneal injection and blood glucose was measured at indicated timepoints thereafter. The results are expressed as mmol/L (**A**,**D**) or pmol/L (**B**). In the statistical analysis, one-way ANOVA and Bonferroni post-test was used in (**A**–**C**) and repeated-measures two-way ANOVA with Bonferroni post-test in (**D**). Mean values significantly different from the HF diet group are marked with * = *p* < 0.05, ** = *p* < 0.01, *** = *p* < 0.001, and **** = *p* < 0.0001. Values are given as the mean + SEM, *n* = 12 mice per group.

The fasting glucose and insulin levels were measured at the end of the study. Interestingly, LSE supplementation significantly prevented the increase in the blood glucose level induced by HF diet, and there was no difference in glucose levels between the HF + LSE and LF diet groups. Fasting blood glucose was 9.1 ± 0.3 mmol/L in the LF diet group, 10.5 ± 0.4 mmol/L in the HF diet group, and 8.9 ± 0.3 mmol/L in the HF + LSE diet group, respectively (*p* < 0.05 between LF vs. HF and HF vs. HF + LSE diet groups) (Figure 2A). HF diet also induced a significant increase in the fasting insulin concentration (*p* < 0.05 between LF vs. HF diet groups). There was not, however, any significant difference in the insulin levels between the HF and HF + LSE diet groups (Figure 2B). Accordingly, HOMA-IR index was significantly higher (*p* < 0.05) in the HF diet group (9.8 ± 1.6) than in the LF diet group (5.4 ± 0.3). In the HF + LSE group, there was a slight trend towards a lower HOMA-IR level as compared to the HF diet group, averaging 8.1 ± 0.8; but the difference was not statistically significant, as seen in Figure 2C.

Glucose tolerance test was carried out at week five, one week before the end of the study. Glucose was given intraperitoneally to the mice after a six-hour morning fast, and blood glucose levels were analyzed before and 30, 60, 120 and 180 min after the glucose injection (Figure 2D). Glucose tolerance was significantly disturbed by the HF diet, which was evidenced by the increased glucose peak at 30 min and by the retarded return to basal level. LSE did not alter the peak value at 30 min, but glucose levels returned faster to the pre-glucose levels in the HF + LSE diet group than in the HF diet group (*p* < 0.05 at 60 min and *p* < 0.01 at 120 ja 180 min), indicating a less severe glucose intolerance in the LSE-treated group.

### 3.4. Cholesterol, Triglycerides, and Adipokines

HF diet induced a significant increase in the serum cholesterol levels when compared to the LF diet group (*p* < 0.001 between the groups), but there was no difference in triglyceride levels. LSE supplementation did not affect either cholesterol or triglyceride levels (Table 1). Serum concentrations of the adipokines adiponectin, leptin, and resistin were also measured. HF diet induced a decrease in the adiponectin and an increase in the leptin levels when compared to the LF diet group (*p* < 0.01 and *p* < 0.001, respectively). There was no difference in the adipokine levels between the HF + LSE and HF diet groups (Table 1).

## 4. Discussion

Our main finding in this study was that lingonberry skin extract prevented the HF diet-induced increase in the fasting glucose level and body weight gain in mice. In addition, the amount of epididymal fat was lower in the lingonberry-supplemented group when compared to the HF diet control group. The results support our previous results, where air-dried lingonberry powder had beneficial metabolic effects in HF diet-induced obesity in mice [8]. Furthermore, the present study extends the previous data on antidiabetic effects of lingonberry, as we now also performed an intraperitoneal glucose tolerance test (IPGTT). The IPGTT test showed a less severe glucose intolerance in lingonberry skin extract-supplemented mice than in the control HF diet-fed mice.

HF diet has been reported to cause insulin resistance, glucose intolerance, increased blood glucose levels, and diabetes [27,28]. Lingonberry skin extract supplementation partly prevented these effects. Importantly, HF diet-induced increase in fasting glucose levels were prevented by LSE-supplementation. In addition, glucose intolerance as assessed by IPGTT was less severe in mice on the lingonberry skin extract-supplemented diet. This was evidenced by the finding that although the peak serum glucose levels were increased, they returned to the basal level faster in the HF + LSE group than in the control HF diet group. Possibly, lingonberry skin extract retarded the development of HF diet-induced insulin resistance, which could explain the normal blood glucose levels, similarly increased insulin levels, and less severe glucose intolerance in IPGTT in the LSE + HF diet group when compared to the control HF diet group. We also calculated the HOMA-IR value, which is an index developed to evaluate the degree of insulin resistance from fasting insulin and glucose levels [17,29]. In the present study, HOMA-IR levels were expectedly increased in the HF diet group. In the HF + LSE group there was a trend towards lower levels in the average values of HOMA-IR, but the difference was not statistically significant, and thus not supporting the assumption of the preventive effect of the LSE-supplementation on HF-diet induced insulin resistance.

Antidiabetic effects of lingonberry have also been reported elsewhere. Freeze-dried lingonberry supplementation was shown to prevent the HF diet-induced rise in fasting glucose levels in mice [10]. In a clinical study, lingonberry improved postprandial glycemic profiles after the consumption of a sucrose-sweetened lingonberry meal, compared to a similar meal without lingonberry [30]. In an in vitro study, lingonberry extract stimulated basal and insulin-stimulated glucose uptake by skeletal muscle cells (C2C12 cell line) and adipocytes (3T3-L1 cell line) [31]. Proposed mechanisms of the antidiabetic effects of berry polyphenols include the stimulation of insulin secretion, increase in glucose uptake by the AMP-activated protein kinase (AMPK) pathway, and inhibition of starch-degrading polysaccharides [14]. Possible compounds identified in lingonberry, which could affect glucose metabolism, are proanthocyanidins [32], quercetin [33], and resveratrol [34].

Weight gain during the HF diet was retarded by lingonberry skin extract supplementation in the present study. This supports our previous study where air-dried lingonberry powder had similar effects [8]. In another study with freeze-dried lingonberries, a similar effect was seen [10]. Also, even a short-time (4 days) lingonberry feeding of mice prevented HF diet-induced weight gain [35]. However, in some other studies, lingonberry supplementation of an HF diet had only a minor or no effect on weight gain [36,37]. These differences may arise from factors, such as the fat content of the HF diet; all other studies cited above used HF diet containing 45% energy from fat, but the study by Huang et al. [36], in which no effect on weight gain was found, used an HF diet containing 60% energy from fat. Huang et al. [36] also used a lower level of lingonberry supplementation (6%), as the other studies had lingonberry supplementation of 20% *w*/*w*. This suggests that the lower lingonberry dose in the study by Huang et al. [36] was not enough to overcome the detrimental effects of the HF diet with 60% energy from fat. Furthermore, natural variation in the content of the bioactive compounds in the lingonberry material has also been found to explain different results in similar study settings [37].

Importantly, epididymal fat gain was also significantly lower in the HF + LSE diet group when compared to the control HF diet group. Lingonberry skin extract supplementation thus partly prevented the HF diet-induced epididymal fat gain. Epididymal (visceral) adipose tissue is known to be specifically detrimental to metabolic health and cause low-grade inflammation in the body, while subcutaneous adipose tissue is considered more neutral or less detrimental [38].

Leptin is an adipokine which regulates appetite and energy metabolism, aiming to prevent weight gain in normal (lean) conditions. Obesity typically increases circulating leptin levels, but the effects of leptin may be decreased due to developing leptin resistance. This results in further increased leptin levels without the full effects of leptin to retard weight gain [39,40]. This phenomenon was also seen in this study, as increased leptin levels were measured in both HF diet groups. The concentration of leptin closely relates to the amount of adipose tissue and body mass index [39]. In the present study, circulating leptin concentrations in the HF + LSE group at the end of the study were at the same level as those in the HF diet group, while the weight of the mice was lower in the HF + LSE group. One possible explanation to this seemingly contradictory result is that lingonberry skin extract may increase leptin secretion and that in turn may contribute to the finding that the lingonberry-supplemented mice were leaner. Another possible explanation to this outcome could be that lingonberry skin extract may enhance the effects of leptin, possible due to less severe leptin resistance; this in turn may contribute to the finding that despite similar leptin concentrations, weight gain in the HF + LSE diet group was lower than that in the control HF diet group. However, further studies are warranted to find out the actual mechanisms behind how lingonberry skin extract retards weight gain induced by an HF diet.

We also measured the levels of two other adipokines: adiponectin and resistin. Adiponectin is known for its anti-inflammatory and insulin-sensitizing properties, and its level reduces in obesity [41,42]. Decreased adiponectin concentrations in the HF diet group were also seen in the present study. There was, however, no difference in the adiponectin levels between the two HF diet groups; even the weight was lower in the HF + LSE diet group. Resistin is a proinflammatory adipokine secreted from adipose tissue, and in mice, it has been connected to insulin sensitivity and insulin resistance [43,44]. In the present study, resistin concentrations were at the same level in all three diet groups with no significant differences indicating an absence of resistin-mediated responses.

In our previous study, whole lingonberry was used as an air-dried powder, containing concentrated lingonberry juice solids, berry skin, and seeds [8]. In the present study, lingonberry seeds were removed from the press cake before the skin extract was prepared. That may explain, at least partly, the finding that LSE had no effect on blood lipids, contrary to our previous results with air-dried lingonberry powder. The lingonberry seed oil consists almost entirely of unsaturated fatty acids [45,46], which are considered beneficial for cholesterol level [47]. Lingonberry juice contains a variety of phenolic compounds, most abundantly procyanidins and anthocyanins [48]. Thus, we conclude that lingonberry compounds affecting lipid metabolism are different from those which affect glucose level, with the former being present in juice and/or seeds of the berry.

Our aim was to find out if a side stream material from berry juice industry (pomace) has any potentially beneficial metabolic properties. The results suggest that berry skin material from the lingonberry juice process could have beneficial effects on glucose metabolism if modified/processed into an easily edible form. Our results are supported by other studies which also used lingonberry press residue as a raw material for extract production: purified polyphenol–polysaccharide conjugates prevented the weight gain of high-cholesterol fed hamsters [49], and elsewhere, the lingonberry pomace extract showed hypoglycemic properties in vitro as it exerted inhibitory effects on α-amylase and α-glucosidase activity [50].

In conclusion, lingonberry skin extract supplementation was found to prevent the rise in fasting glucose level, and also weight and visceral fat gain in a mice model of HF diet-induced obesity. The results extend previous data of beneficial metabolic effects of lingonberry and encourage the use of side streams of lingonberry juice process in health-promoting applications. Further studies on the topic are warranted, also clinically.

## Figures and Tables

**Table 1:** Cholesterol, triglyceride, and adipokine levels in mice at the end of the study.

	Low-Fat (LF) Diet	High-Fat (HF) Diet	High-Fat + Lingonberry (HF + LSE) Diet	*p*-Value between LF and HF Diet Groups	*p*-Value between HF and HF + LSE Diet Groups
Cholesterol (mmol/L)	1.9 ± 0.1	2.7 ± 0.1	2.7 ± 0.2	***	ns
Triglycerides (mmol/L)	0.8 ± 0.1	0.7 ± 0.1	0.7 ± 0.1	ns	ns
Adiponectin (µg/mL)	9.2 ± 0.4	7.6 ± 0.2	7.8 ± 0.3	***	ns
Leptin (ng/mL)	7.0 ± 0.7	23.1 ± 2.6	22.1 ± 1.6	***	ns
Resistin (ng/mL)	21.2 ± 1.4	20.2 ± 1.0	23.6 ± 1.7	ns	ns

Animals were given low-fat diet (LF diet, 10% of energy from fat) or high-fat diet (HF diet, 46% of energy from fat) for six weeks. Mice were dosed each weekday with vehicle (LF and HF diet groups) or lingonberry skin extract (HF + LSE diet group) 8.5 µL/g body weight by intragastric administration. Blood samples were collected after 6 h of fasting, and serum cholesterol and triglyceride concentrations were analyzed with fluorometric assays. Adiponectin, leptin, and resistin concentrations were measured with ELISA. Values are presented as the mean ± SEM, with *n* = 12 mice in each group. One-way ANOVA with Bonferroni post-test was used in the statistical analysis. Differences between the groups are marked with *** = *p* < 0.001, and ns = not significant.

## Data Availability

All relevant data are within the paper.

## Supplementary Materials

The following supporting information can be downloaded at: https://www.mdpi.com/article/10.3390/nu16132107/s1, A representative figure of each phenolic compound group analyzed with HPLC.

## Abbreviations

AMPK	AMP-activated protein kinase
C2C12	Murine skeletal myoblast cells
HF	High-fat
LF	Low-fat
LSE	Lingonberry skin extract
ORAC	Oxygen radical absorbance capacity
IPGTT	Intraperitoneal glucose tolerance test
3T3-L1	Murine preadipocyte cells.
