# Supplementary material for: Lingonberry (Vaccinium vitis-idaea L.) Skin Extract Prevents Weight Gain and Hyperglycemia in High-Fat Diet-Induced Model of Obesity in Mice
Source: Nutrients. 2024 Jul 2;16(13):2107. doi: 10.3390/nu16132107 (PMC11243352; doi:10.3390/nu16132107)
Supplement: Supplementary file 1 [file nutrients-16-02107-s001.zip › nutrients-2994018-supplementary.pdf]

Supplementary material

Representative HPLC grams of the analysed compound groups. The methods are described in the chapter 4.2 in the Materials and Methods.

A. <u>Phenolic acids</u>

Gallic acid (1), protocatechuic acid (2), p-hydroxybenzoic acid (3), chlorogenic acid (4), vanillic acid (5), caffeic acid (6), syringic acid (7), p-coumaric acid (8), ferulic acid (9), sinapinic acid (10), benzoic acid (11) and cinnamic acid (12)

λ 260 nm (quantitatively analysed acids: 1-3, 5, 7, 11-12)

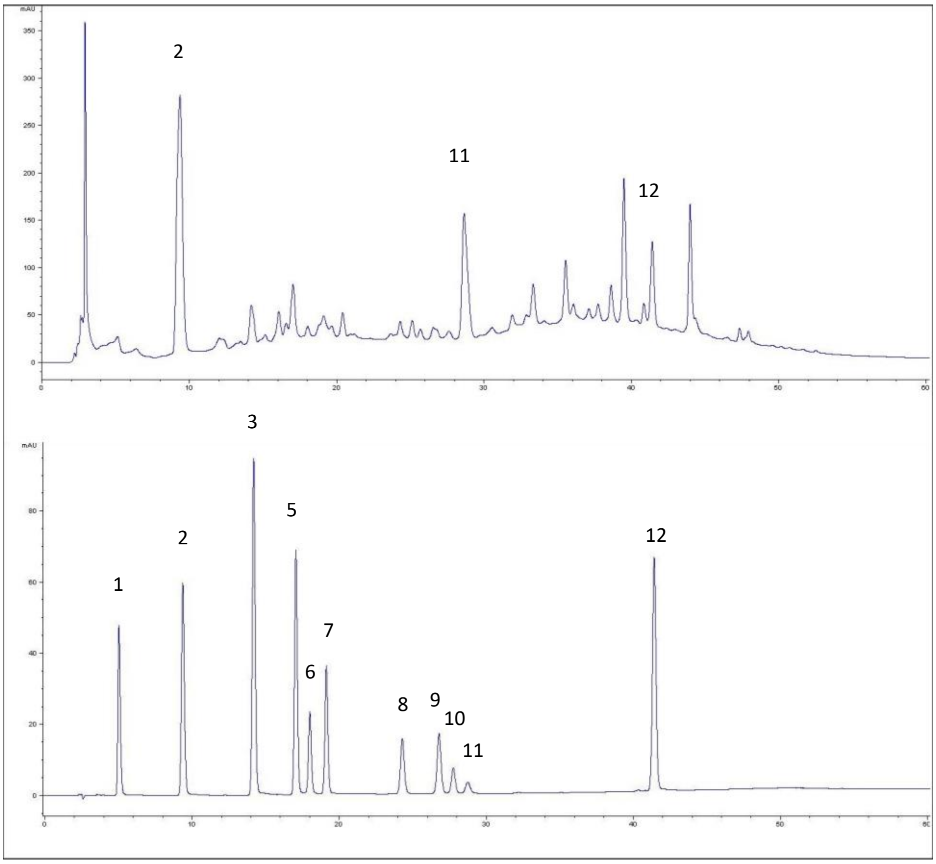

The sample is in the upper figure, and standard mixture (concentration 6,3 µg/mL) in the lower figure.

Supplementary material

λ 320nm (quantitatively analysed acids: 4, 6, 8-10)

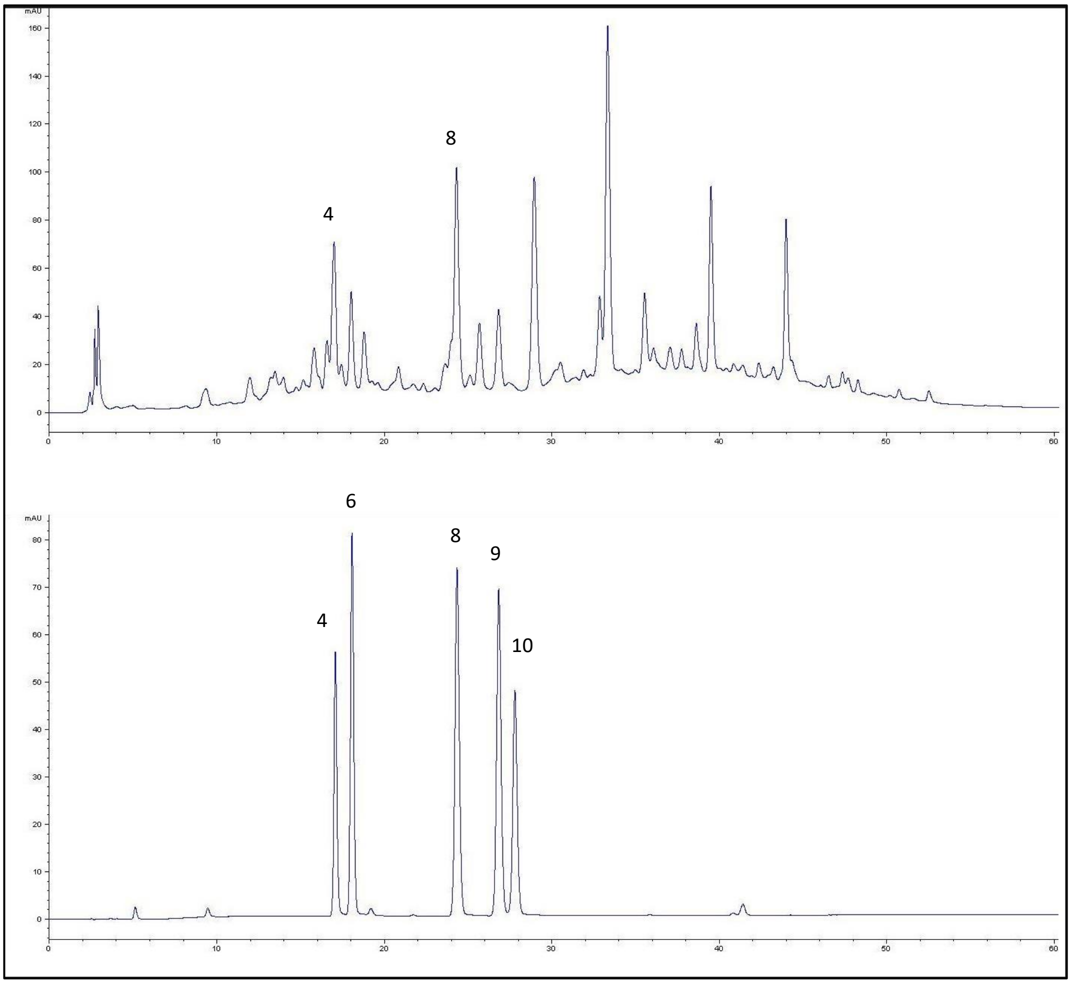

The sample is in the upper figure, and standard mixture (concentration 6,3 μg/mL) in the lower figure.

Supplementary material

B. Quercetin

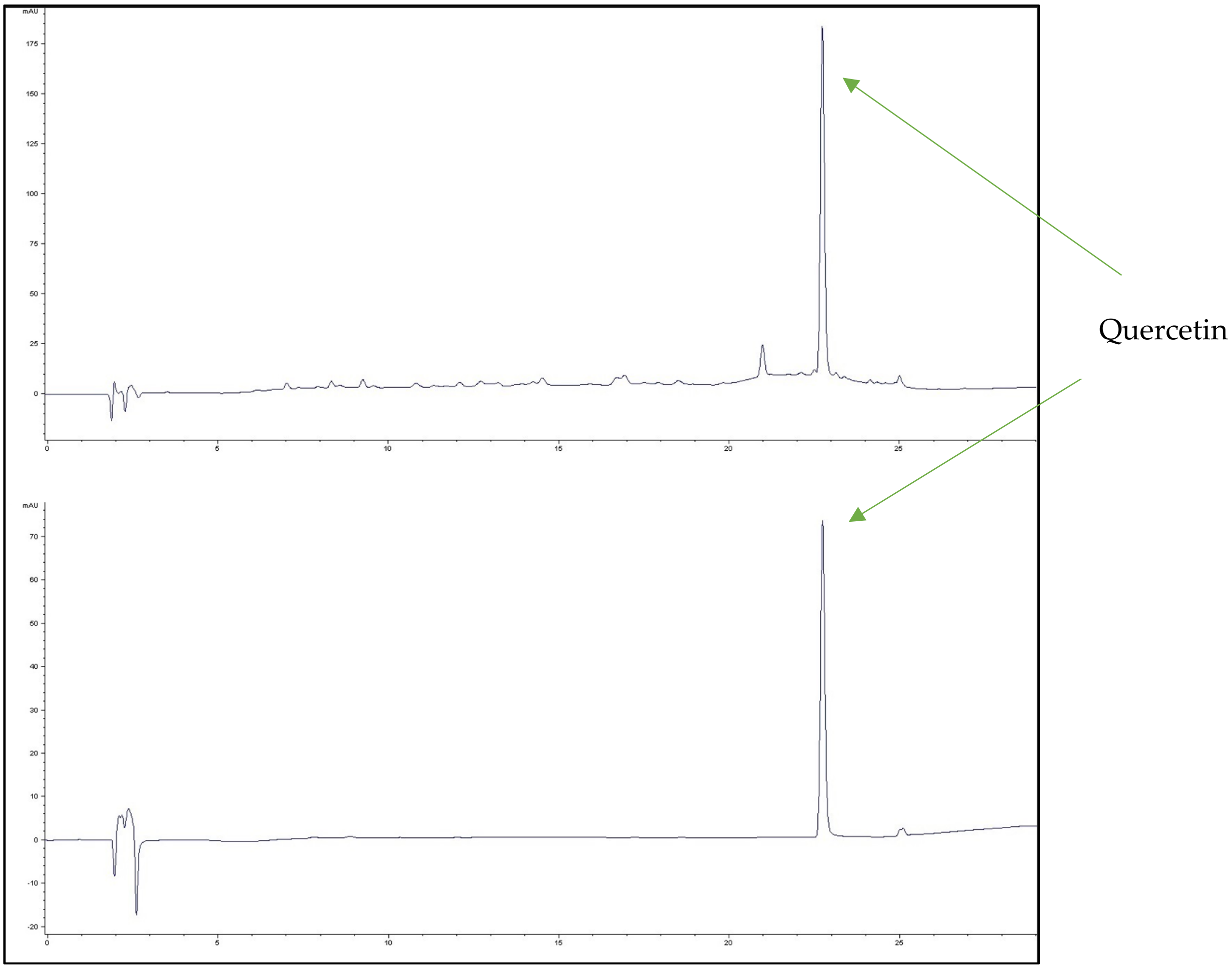

The sample is hydrolyzed and contains both free quercetin and quercetin glycosides (the upper figure). Quercetin standard mixture (concentration 6,9 μg/mL) is in the lower figure.

Supplementary material

C. Anthocyanins

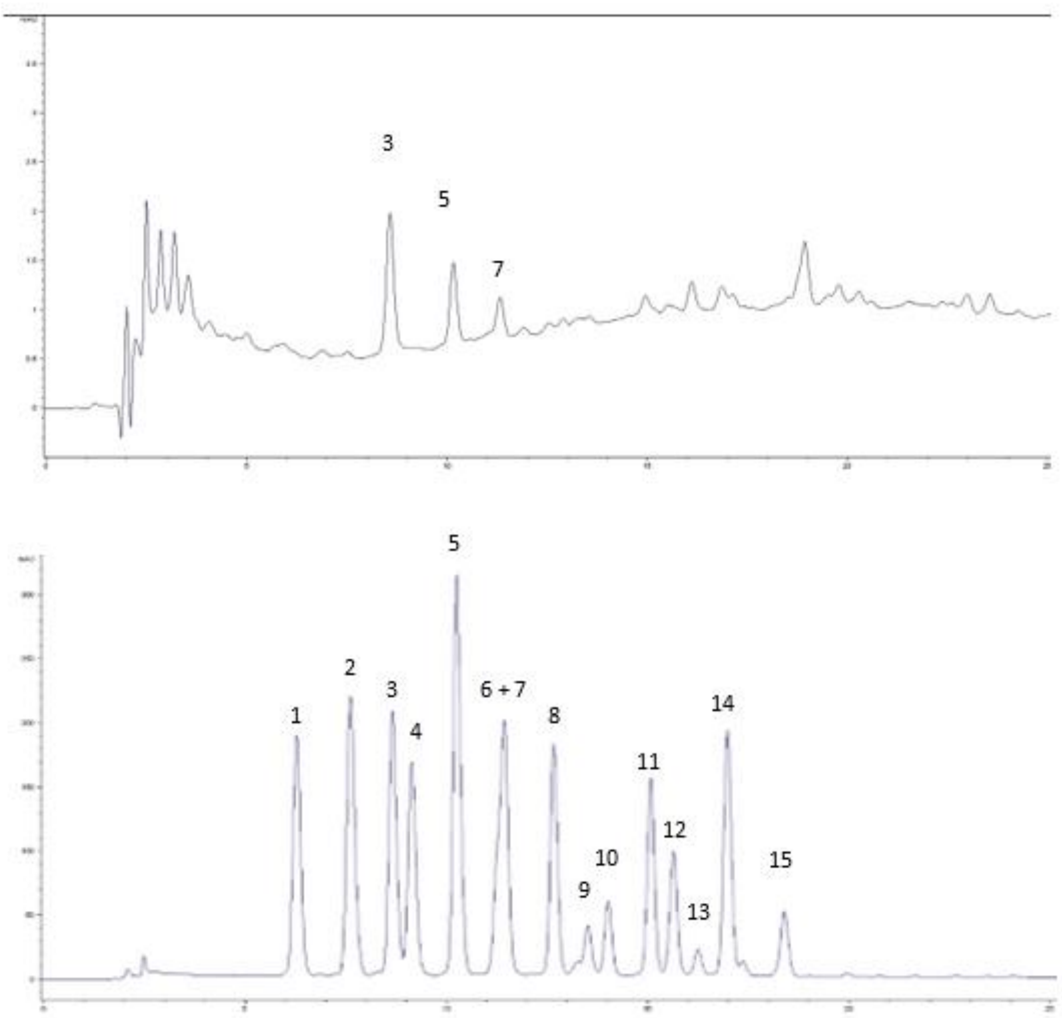

The sample is in the upper figure, and in-house bilberry juice control for anthocyanins in the lower figure. Explanation of numbers: Delphinidin-3-galactoside (1), delphinidin-3-glucoside (2), cyanidin-3-galactoside (3), delphinidin-3-arabinoside (4), cyanidin-3-glucoside (5), petunidin-3-galactoside (6), cyanidin-3-arabinoside (7), petunidin-3-glucoside (8), peonidin-3-galactoside (9), petunidin-3-arabinoside (10), peonidin-3-glucoside (11), malvidin-3-galactoside (12), peonidin-3-arabinoside (13), malvidin-3-glucoside (14) and malvidin-3-arabinoside (15).
